# ASIC1 promotes differentiation of neuroblastoma by negatively regulating Notch signaling pathway

**DOI:** 10.18632/oncotarget.14164

**Published:** 2016-12-24

**Authors:** Mingli Liu, Koichi Inoue, Tiandong Leng, An Zhou, Shanchun Guo, Zhi-gang Xiong

**Affiliations:** ^1^ Department of Microbiology, Biochemistry & Immunology, Atlanta, GA 30310, USA; ^2^ Neuroscience Institute, Morehouse School of Medicine, Atlanta, GA 30310, USA; ^3^ Department of Chemistry, RCMI Cancer Research Center, Xavier University of Louisiana, New Orleans, LA 70125, USA

**Keywords:** ASIC1a, Notch, NS20Y, neurite growth

## Abstract

In neurons, up-regulation of Notch activity either inhibits neurite extension or causes retraction of neurites. Conversely, inhibition of Notch1 facilitates neurite extension. Acid-sensing ion channels (ASICs) are a family of proton-gated cation channels, which play critical roles in synaptic plasticity, learning and memory and spine morphogenesis. Our pilot proteomics data from ASIC1a knock out mice implicated that ASIC1a may play a role in regulating Notch signaling, therefore, we explored whether or not ASIC1a regulates neurite growth during neuronal development through Notch signaling. In this study, we determined the effects of ASIC1a on neurite growth in a mouse neuroblastoma cell line, NS20Y cells, by modulating ASIC1a expression. We also determined the relationship between ASIC1a and Notch signaling on neuronal differentiation. Our results showed that down-regulation of ASIC1a in NS20Y cells inhibits CPT-cAMP induced neurite growth, while over expression of ASIC1a promotes its growth. In addition, down-regulation of ASIC1a increased the expression of Notch1 and its target gene Survivin while inhibitor of Notch significantly prevented the neurite extension induced by ASIC1a in NS20Y cells. These data indicate that Notch1 signaling may be required for ASIC1a-mediated neurite growth and neuronal differentiation.

## INTRODUCTION

The Notch signaling pathway is critical for cell fate determination and function both in the embryonic brain and adult brain [1–2]. Activated Notch1 maintains neural stem cell characteristics, induces proliferation, and inhibits both neural stem cells (NSCs) and more limited intermediate neural progenitors (INPs) differentiation. Abrogation of Notch signaling *in vivo* and *in vitro* promotes neurogenesis with a transition from neural stem or precursor cells to transient-amplifying cells or neurons [3–7]. In neurons, it has been shown that up-regulation of Notch1 activity either inhibited neurite extension or caused retraction of neurites. Conversely, inhibition of Notch1 signaling facilitated neurite extension [8–9]. Neurite growth is required for nervous system development and repair. Cerebral cortical neurons grow by extending neurites (axons and dendrites) and form connections as neurons mature. Acid-sensing ion channels (ASICs) are a family of proton-gated cation channels and regulate synaptic physiology. They contribute to neuronal injury associated with neurological disorders such as brain ischemia, multiple sclerosis, and spinal cord injury [10–14]. Recently, a good correlation has been found between ASIC1a expression and spine density [15], suggesting that ASICs also play essential roles in spine morphogenesis, maintenance and remodeling. Degenerin/epithelial Na+ channels (DEG/ENaC) are found to be required for nerve growth factor (NGF)-induced neurite growth [16]. However, whether ASIC1, another member of DEG/ENaC [17–19], regulates neurite growth remains elusive. In a pilot quantitative proteomic analysis of WT and ASIC1a knockout mouse brains (unpublished data), we found that lacking ASIC1a is associated with a decrease in proteins involved in Notch signaling. To further define the role of ASIC1a in neuronal remodeling and differentiation, we determined whether or not ASIC1a regulates neurite growth through Notch signaling during neuronal development. NS20Y cell line, a mouse cholinergic neuroblastoma, was commonly used for determining neurite growth [25–27]. The NS20Y was adapted to undifferentiated growth in suspension culture while underwent differentiation by transferred to surface culture and treated with a variety of reagents including 8-(4-chlorophenylthio) adenosine 3’,5’-cyclic monophosphate (8-CPT-cAMP or CPT-cAMP) [28–29], retinoic acid or serum [30–32]. The NS20Y cell differentiation has crucial features which have been seen in normal neuronal development providing an appropriate model for investigating neuronal development. In addition, the NS20Y, a clonal population cells provides a great advantage for molecular studies [30–32]. Therefore, in the present study, we determined the effect of ASIC1a on neurite growth using NS20Y cell line.

## RESULTS

### Down-regulation of ASIC1a in NS20Y cells inhibits CPT-cAMP-induced neurite growth, while over expression of ASIC1a promotes its growth

**Figure 1:**
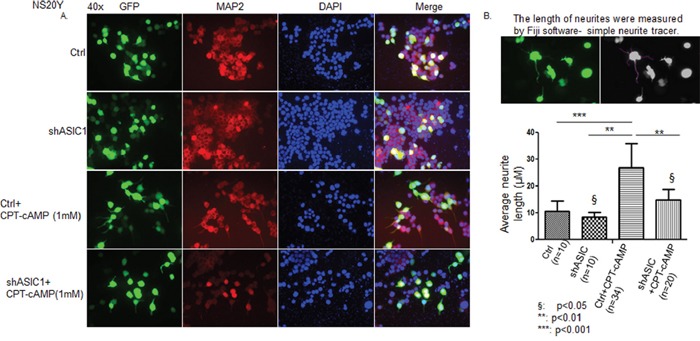
Down regulation of ASIC1a in NS20Y cells reduced CPT-cAMP-induced neurite growth NS20Y cells were transfected with a short hairpin ASIC1a (sh ASIC1a) or a control vector tagged with GFP, cells were left untreated or treated with 1 mM CPT-cAMP for 72h. After fixation the cells were probed with antibodies as indicated and photographed at 40x using fluorescecnt microscope. **A.** The parental NS20Y cells are round and spindle shaped, there is no dendrite on the cell body. After treated with 1 mM CPT-cAMP, cells showed polygonal shaped cells with dendrites all over the cell body. All cells were MAP2 positive by double immunostaining, implying that they were neuronal origin. **B.** Quantitative measurement of neurite lengths using Simple Neurite Tracer software (upper panel) showed that average neurite length was 2.6-fold of control in cells treated with CPT-cAMP (n=34), while this increase was reduced by shASIC1a to only 1.4-fold of control (n=20) (lower panel).

**Figure 2:**
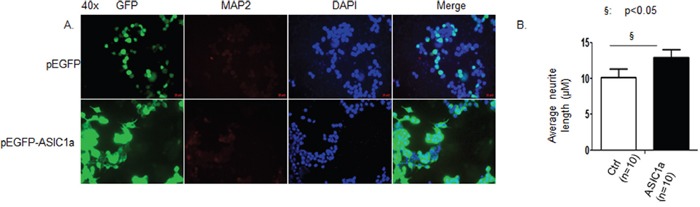
Over expression of ASIC1a promotes neurite growth NS20Y cells were transfected with a plasmid overexpressing ASIC1a (pEGFP-ASIC1a). After 72h, the cells were fixed and probed with antibodies as indicated and photographed at 40x using fluorescecnt microscope. **A.** NS20Y cells are round and spindle shaped, there is no dendrite on the body of the cell. When overexpressed with ASIC1a, cells showed polygonal shaped cells with dendrites all over the cell body. All cells were MAP2 positive by double immunostaining, indicating that they were neuronal origin. **B.** Quantitative measurement of neurite lengths using Simple Neurite Tracer showed that average nurite length of NS20Y cells increased to 1.29-fold of control when they were overexpressed with ASIC1a.

NS20Y cells were plated at approximately 70% confluence. After 24 h cells were either transfected with a short hairpin ASIC1a (sh ASIC1a) or a control vector with both vectors tagged with GFP, then cells of each group were left untreated or treated with 1 mM CPT-cAMP. After 72 h, cells were fixed and probed with the antibodies as indicated and photographed at 40x using fluorescent microscope. As shown in Figure 1, the undifferentiated NS20Y cells are round and spindle shape; there are no clear dendrites on the body of the majority of cells (Figure 1A). When treated with 1 mM CPT-cAMP, NS20Y cells showed polygonal shape and had many dendrites on the cell body. Only 2-4% of cells had neurites greater than the length of the cell body in controls, while 15-20% of CPT-cAMP-treated cells had extended neurites. Double staining experiments demonstrated that all transfected cells were neuronal in origin, as assessed by positive MAP2 immunostaining (Figure 1A). Quantitatively measuring neurite lengths by Simple Neurite Tracer (Figure 1B upper panel), we found that average neurite length in cells treated with CPT-cAMP increased 2.6-fold of control. This increase was however reduced by shASIC1a to only 1.4-fold of control (Figure 1B lower panel). In contrast, when ASIC1a was overexpressed in NS20Y cells, the average neurite length increased to 1.29-fold of control (Figure 2A and 2B). These results indicated an important role for ASIC1a in promoting neurite growth.

### Down-regulation of ASIC1a by shRNA increases while up-regulation of ASIC1a decreases *Notch1* and its target gene *Survivin* in NS20Y cells

**Figure 3:**
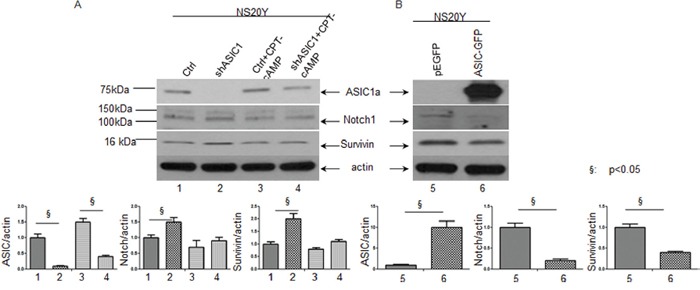
Down regulation of ASIC1a by shRNA increases while up regulation of ASIC1a decreases Notch1 and its target gene Survivin in NS20Y cells The shASIC1a and pEGFP-ASIC1a constructs were transfected into NS20Y cells and the changes in expression levels of Notch1signaling were then determined by Western blot. **A.** The levels of endogenous expression of Notch1 and its downstream targeting gene, Survivin were detected in NS20Y cells. Both Notch1and Survivin are constitutively expressed in NS20Y (lane 1 and 5 in upper panel), knock-down of shASIC1a resulted in upregulation of Notch1 gene and its downstream target Survivin gene (upper left panel, lane 1 vs. 2 and lane 3 vs.4); **B.** While overexpression of ASIC1a downregulated Notch1 and Survivin genes (upper right panel).

**Figure 4:**
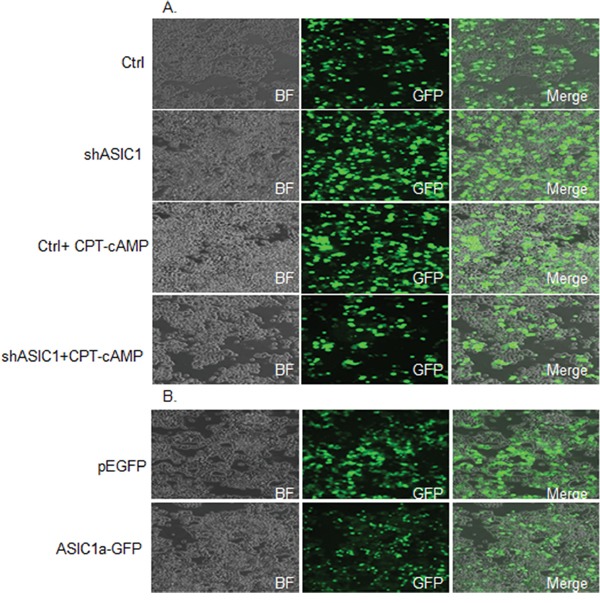
The transfection efficiency is around 70-80% as shown by the ratio of GFP-positive cells to the total number of cells in different groups **A.** NS20Y cells in contral group and those treated with shASIC1a, CPT-cAMP, and shASIC1 plus CPT-cAMP. **B.** NS20Y cells overexpressed with ASIC1a-GFP. Cells treated with control vector pEGFP were also shown.

**Figure 7:**
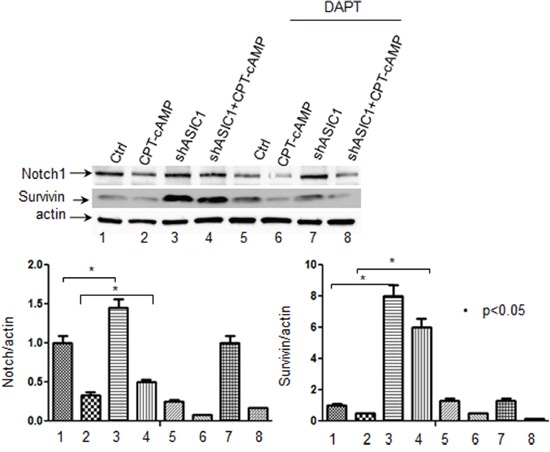
The effectiveness of DAPT was confirmed by reduced protein levels of Notch1 and its responsive gene Survivin upon treatment of DAPT in NS20Y cells DAPT effectively inhibited the Notch response by causing a reduction on the level of Notch1 and its responsive gene Survivin (lane 1,2,3,4 vs.5,6,7,8 respectively).

Notch signaling plays a crucial role in the development of vertebrate nervous system [33]. The activation of Notch signaling pathway inhibits cellular differentiation including neurite outgrowth [34]. Among several Notch receptors, Notch1 signaling is an evolutionarily conserved pathway crucial for the development and homeostasis of many organs. The critical roles of Notch1 have been observed extensively during tumorigenesis and prognosis in a variety of cancers. For instance, Notch1 was showed to be linked to hepatocellular carcinoma development, tumor recurrence and invasion, which is mediated in part through the Notch1-E-Cadherin pathway [35]. Notch1 pathway-mediated microRNA-151-5p promotes gastric cancer progression [36]. Down regulation of Notch1 signaling pathway can sensitize some anticancer drug (s) in trastuzumab-resistant breast cancer cells [37]. In diagnostic and clinical trial cohorts, patients suffered from chronic lymphocytic leukemia with Notch1 coding mutations or noncoding mutations exhibited reduced survival [38–39]. In addition, our proteomics data showed that ASIC1a is a regulator of Notch. We therefore determined whether ASIC1a is a key regulator of Notch1 gene expression during neuronal differentiation. We first examined the levels of endogenous expression of Notch1 and its downstream targeting gene, Survivin in NS20Y cells. We found that both Notch1 and Survivin are constitutively expressed in NS20Y cells as shown by Western blot analysis in Figure 3A (lane 1) and Figure 3B (lane 5). Next, we transfected shASIC1a and pEGFP-ASIC1a constructs into NS20Y cells and then determined the changes in expression levels of Notch1signaling by Western blot. The ASIC1a protein is undetectable in Western blot when ASIC1a was knock-down by shASIC1a in Figure 3A (lane 1 vs. lane 2), indicating the high transfection efficiency. More intriguingly, overexpression of ASIC1a led to 10 times up-regulation of ASIC1a proteins as shown in Figure 3B (lane 5 vs lane 6). Similarly, GFP-positive cells in immunofluorescence staining images confirmed the high transfection efficiency to be about 70-80%; the representative images were shown in Figure 4A and 4B demonstrating GFP-positive cells as compared to the total number of cells shown in the bright field (BF). When the relationship between ASIC1and Notch1 was determined, c ontrary to the proteomics data shown in Table 1, knock-down of ASIC1a by shASIC1a resulted in up-regulation of Notch1 gene and its downstream target Survivin gene (Figure 3A lane 1 vs. 2 and lane 3 vs.4; also see Figure 7 lane 1vs.3 and lane 2 vs.4, lane 5 vs.7, lane 6 vs.8), while overexpression of ASIC1a downregulated Notch1 and Survivin genes (Figure 3B).

### DAPT, an inhibitor of Notch1 significantly abolishes inhibition of neurite extension by downregulating ASIC1a in NS20Y cells

**Figure 5:**
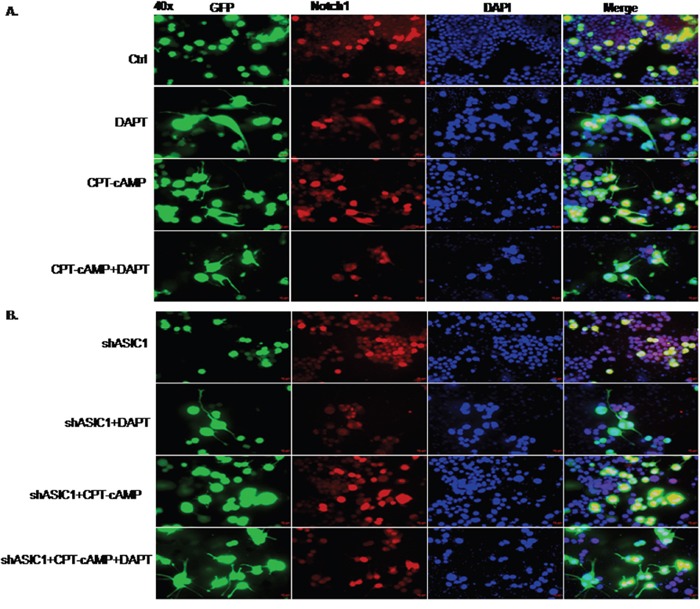
DAPT, an inhibitor of Notch1 significantly abolishes inhibition of neurite extension in NS20Y cells To test whether the ASIC1a and Notch molecules act synergistically or dependently upon neuronal differentiation, we inactivated Notch signaling by DAPT, a commonly used Notch inhibitor, and then tested the effect of ASIC1a signaling on neuronal differentiation and neurite growth. NS20Y cells were transfected with shASIC1a for 24h, and treated with DAPT (50 nM) for 48h. Morphologically, average neurite length was increased after the cells were treated with DAPT in different groups: **A.** ctrl vs DAPT, CPT-cAMP vs. CPT-cAMP+DAPT; **B.** shASIC1 vs shASIC1+DAPT, shASIC1+CPT-cAMP vs shASIC1+CPT-cAMP+DAPT

**Figure 6:**
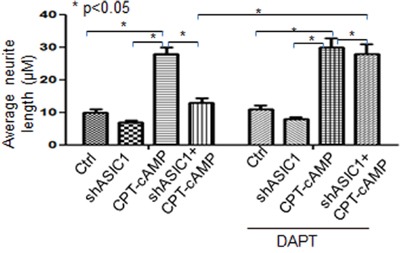
Quantitative measurement of neurite lengths using Simple Neurite Tracer of data from Figure 5 The effects of DAPT on the morphological changes of neurite were more significant when pre-treating cells with shASIC1a by which neurite growth was depressed (compare shASIC1+CPT-cAMP to shASIC1+CPT-cAMP+DAPT).

During the neurite outgrowth, inactivation of Notch1 and activation of the ASIC1a resulted in neurite extension. We wondered whether the ASIC1a and Notch1 molecules function integrately or dependently. To this end, we inactivated Notch1 by DAPT (N-[N-(3,5-difluorophenacetyl)-l-ananyl]-*S*-phenylglycine *t*-butyl ester), a typical Notch1 inhibitor, followed by testing how changes in Notch1 activity affect neuronal differentiation and neurite growth induced by ASIC1a. Typically, DAPT prevents full-length Notch1 from cleavage by the presenilin-γ-secretase complex to generate Notch1 intracellular domain (NICD). Failure to release of Notch1 intracellular domain leads to inactivation of Notch1 [33]. In the present study, NS20Y cells were transfected with shASIC1a for 24h followed by treatment with DAPT (50 nM) for 48h. The effectiveness of DAPT was confirmed by reduced protein levels of Notch1 and its responsive gene Survivin upon treatment of DAPT (Figure 7, lane 1,2, 3, 4 vs.5,6,7,8 respectively, not shown in the Figure 7 for clarity). Our results showed that the average length of neurites was increased after the cells were treated with DAPT (Figure 5A, 5B). Notably, these morphological changes were more significant when pre-treating cells with shASIC1a (Figure 5B and Figure 6, shASIC1+CPT-cAMP vs. shASIC1+CPT-cAMP+DAPT). These data indicated that Notch1 signaling is functioning downstream of ASIC1a, Notch1 mediated the inhibition of neurite growth by reduced level of ASIC1a. In another word, ASIC1a is signaling through the Notch pathway to affect neurites growth.

## DISCUSSION

Acid-sensing ion channels (ASICs) are proton-gated amiloride-sensitive cation channels widely expressed in neurons of peripheral sensory and central nervous system [40]. Activation of ASICs plays an important role in physiological processes such as nociception, mechanosensation, synaptic plasticity, learning and memory. ASICs also contribute to some pathological processes such as brain ischemia, multiple sclerosis, traumatic neuronal injury, Parkinson's disease [41–42], epileptic seizures [43] and psychiatric diseases. Upon immunostaining and mRNA assay in the rat brain, ASIC1 has been found broadly distributed in adult brain including the cerebral cortex, hippocampus, cerebellum, olfactory bulb, basolateral amygdaloid nuclei, and subthalamic nuclei [44–45]. The ontogeny of ASIC1a in mouse brain revealed ASIC1a protein is expressed as early as at embryonic day12, and stays constantly to the postnatal period during brain development [46]. The widespread subcellular localization and long term expression during normal brain development emphasize the crucial roles of ASIC1a in neuronal development. Neurons are highly polarized cells with axonal and dendritic domains conveying flux of signaling in the nervous system. Unfortunately, the mechanism by which ASIC1a regulates the precise events such as axo-dendrite formation and afterwards neurite elongation still remain poorly understood. Our results in the present study showed that down-regulation of ASIC1a in NS20Y cells inhibits CPT-cAMP induced neurite growth, while over expression of ASIC1a promotes its growth.

Notch signaling plays an important role in cell fate decisions in uncommitted proliferative cells during neuronal development. The Notch signaling pathway is well known to maintain characteristics of neural stem cells by inhibition of neurogenesis [47]. Notch also inhibits neurite outgrowth and therefore influences neurite morphology in postmitotic neurons [34]. Neurons with low Notch1 expression readily extend neurites, up-regulation of Notch in these neurons inhibit or retract neurite extension [8]. In addition to the roles during neuronal development, Notch signaling regulates migration, morphology, synaptic plasticity and survival of immature and mature neurons [48].

Survivin, the smallest member of the inhibitor-of-apoptosis (IAP) family [49], is the major target of Notch1. It plays an important role in inhibiting apoptosis/mitotic catastrophe, regulating spindle assembly and chromosomal passenger targeting for a centromere checkpoint (CPC) [50]. The agents shutting down the expression of Survivin gene have successfully entered the clinical trials as anticancer strategies [51]. As a downstream target of Notch, Survivin regulates both physiological and pathological process. Notch1 inhibited apoptosis of human pulmonary arterial endothelial cell (hPAECs) via downregulation of Survivin and Bcl-2 and increased proliferation via p21 [52]. During breast oncogenesis, HER2 stabilizes Survivin while concomitantly down-regulating Survivin gene transcription by suppressing Notch1 cleavage [53]. Notch1 signaling is involved in the upregulation of Survivin expression in lung cancer cells as well, which is synergized by HIF-1α [54]. Moreover, crossstalk exists between upstream and downstream molecules of Notch1 intracellular domain (N1ICD). In the present study, we provided the evidence that ASIC1a negatively regulates Notch1 and its major target gene Survivin expression in NS20Y cells indicating that Notch1 acts as downstream of ASIC1a (Figure 3 and Figure 7). The blockade of Notch1 by DAPT prevented the inhibition of neurite growth by reduced ASIC1a expression (Figure 5-7).

The proteomic analysis by comparison of adult ASIC1a−/− and WT mice implicated that Notch signaling pathways were changed. Our results *in vitro* in NS20Y cells showed the reversed relationship between ASIC1a and Notch1 with up-regulation of ASIC1a or down-regulation of ASIC1a in the cells (Figure 3 and Figure 7). The changes in Notch signaling may differ between cell types [48]. It may not be precise to reach conclusions regarding the potential effect of changes in whole-tissue expression levels of Notch protein or mRNA as there are probably pleiotropic roles for Notch in different cell types. For instance, Notch has profound roles not only in neurons and astroglia, but in other cell types in the brain, such as endothelial cells [55] and oligodendrocytes [56–57]. Another greatest challenge in studying Notch signaling is the inability to predict the consequence of activation due to its extensive crosstalk with other multiple pathways [48], which is determined by multiple factors such as the cell ability to receive Notch activation, cell type, animal age, stimulus type and brain region, epigenetic factors [58] and tissue-specific Notch cofactors [59].

Although we found the role of ASIC1a in the regulation of neurite growth mediated by Notch1 signaling, many work still needs to be done. For instance whether Notch1 is the direct downstream molecule of ASIC1 or there are some other molecules located in between need to be examined. In addition, whether the ionic conduction per se is necessary for this function, or conduction-independent functions [60] contribute to this process warrant further investigation.

## MATERIALS AND METHODS

### Antibodies and reagents

Rabbit polyclonal antibody against ASIC1a was a gift from Dr. Xiangming Zha (University of South Alabama College of Medicine, Mobile, Alabama). Polyclonal rabbit antibody against Survivin was purchased from Cell Signaling Technology, Inc (Danvers, MA). Monoclonal antibody to Notch1 and microtubule-associated protein 2 (MAP2) as well as polyclonal β-actin antibody were obtained from Sigma-Aldrich (St.Louis, Mo). All secondary antibodies used for Western blot were purchased from Calbiochem (La Jolla, CA). Short hairpin ASIC1a (shASIC1a) and control shRNA were purchased from SuperArray Bioscience Corporation (Frederick, MD), each vector contains the shRNA under control of U1 promoter and the green fluorescence protein (GFP) gene, for the enrichment of transiently transfected cells. In detail, SureSilencing short hairpin RNA (shRNA) plasmid for human ACCN2 (ASIC1a, Amiloride-sensitive cation channel 2, neuronal) was designed to specifically knock down the expression of *ASIC1a* gene by RNA interference under transient transfection conditions after performance of the appropriate enrichment. The vector contains the shRNA under control of the U1 promoter and the GFP gene, for the enrichment of transiently transfected cells. The RefSeq accession number (NM_020039) refers to the representative sequence used to design the enclosed shRNA, the insert sequence is: GCCAAGAAGTTCAACAAATCT. The sequence of normal control (NC) is GGAATCTCATTCGATGCATAC. The plasmid overexpressing ASIC1a named pEGFP-ASIC1a was constructed as described previously [61–62]. Briefly, the Rat ASIC1a cDNA was fused with a GFP at the c-terminal and inserted into pcDNA3 [61–62]. DAPT (N-[N-(3,5-difluorophenacetyl)-l-ananyl]-*S*-phenylglycine *t*-butyl ester) was purchased from Sigma-Aldrich (St.Louis, Mo). CPT-cAMP 8-(4-chlorophenylthio) adenosine 3’,5’-cyclic monophosphate (8-CPT-cAMP or CPT-cAMP) was purchased from abcam (Cambridge, UK).

### Cell culture

Mouse neuroblastoma cell line NS20Y was purchased from Sigma-Aldrich and plated on poly-L-lysine-coated petri dishes in Dulbecco's minimum essential medium supplemented with 10% heat-inactivated bovine serum at 37°C in a humidified atmosphere of 5% CO2. NS20Y cells were differentiated with treatment of 1 mM CPT-cAMP.

### Immunoflurescence staining

Cells grown in monolayer cultures were fixed with 4% paraformaldehyde in phosphate-buffered saline (PBS), permeabilized with 0.2% Triton X-100, and blocked with 10% goat serum prior to antibody staining. Specific primary antibodies were added at 1:200 dilution overnight. Fluorescein staining was developed using the Alexa-488 or Alexa 555 fluorescence system (Molecular Probes, Carlsbad, CA). Fluorescent images were collected by using a Zeiss LSM510 confocal microscope, and captured with LSM software, version 2.3 (Carl Zeiss, Wetzlar, Germany). After the images of immunostained cells were captured, the neurite lengths were measured using Simple Neurite Tracer (Fiji/Image J plugin; NIH, Bethesda, MD, USA). Briefly, the slide was moved randomly to adjacent fields and the lengths of the neurites in each cell were counted. Neurite length mean ± SEM was calculated based on three independent replicated experiments (n=3). Images from randomly selected fields were analyzed for each replicate and neurite length measurement was performed for up to 100 cells, which correspond to four to six randomly selected fields.

### shRNA transfection

A scrambled shRNA, with no homology to any known sequence was used as control. NS20Y cells were transfected with 5μg of each specific ASIC1a shRNA or control shRNA using Lipofectamine™ reagent in serum free OptiMEM-1 medium (Invitrogen, Carlsbad, CA) per 35 mm dish according to the manufacture's instruction. After transfection, cells were grown in growth medium for another 72h under each treatment as indicated. All studies were done in triplicates.

### Western blot

Cells were lysed with lysis buffer (50 mM HEPES, 150 mM NaCl, 1.5 mM MgCl2, 1 mM EGTA, 10% glycerol, 1% Nonidet P-40, 100 mM NaF, 10 mM sodium pyrophosphate, 0.2 mM sodium orthovanadate, 1 mM phenylmethylsulfonyl fluoride, 10 μg/ml aproptinin, and 10 μg/ml leupeptin). Samples were separated by SDS/PAGE, and separated proteins were transferred to nitrocellulose membranes and identified by immunoblotting. Primary antibodies were diluted at the ratio of 1:1000 according to manufacturer's instruction. Blots were developed with Supersignal Pico substrate (Pierce). The secondary antibodies included HRP-conjugated anti-rabbit and anti-mouse antibodies were obtained from Calbiochem. A densitometric analysis of the bands was performed with the ImageQuant program (Bio-Rad).

### Statistical analysis

Statistically significant differences were determined using Prism statistical software (Graph Prism 4.03, San Diego, CA). All data are presented as mean ± SEM of at least three independent experiments. For data analysis, T-test or one-way ANOVA with Dunnett's or Bonferroni's post test were applied. Statistical significance was set at *p<0.05, **p<0.01, and ***p<0.001.
